# Integrative analysis of Mendelian randomization and Bayesian colocalization highlights four genes with putative BMI-mediated causal pathways to diabetes

**DOI:** 10.1038/s41598-020-64493-4

**Published:** 2020-05-04

**Authors:** Qian Liu, Jianxin Pan, Carlo Berzuini, Martin K. Rutter, Hui Guo

**Affiliations:** 1 Centre for Biostatistics, School of Health Sciences, The University of Manchester, Manchester, UK; 2 School of Mathematics and Statistics, Xidian University, Xi’an, China; 3 School of Mathematics, Faculty of Engineering and Physical Science, The University of Manchester, Manchester, UK; 4 Division of Endocrinology, Diabetes and Gastroenterology, Faculty of Biology, Medicine and Health, The University of Manchester, Manchester, UK; 5 Manchester Diabetes Centre, Central Manchester University NHS Foundation Trust, Manchester Academic Health Science Centre, Manchester, UK

**Keywords:** Predictive markers, Disease genetics

## Abstract

Genome-wide association studies have identified hundreds of single nucleotide polymorphisms (SNPs) that are associated with BMI and diabetes. However, lack of adequate data has for long time prevented investigations on the pathogenesis of diabetes where BMI was a mediator of the genetic causal effects on this disease. Of our particular interest is the underlying causal mechanisms of diabetes. We leveraged the summary statistics reported in two studies: UK Biobank (N = 336,473) and Genetic Investigation of ANthropometric Traits (GIANT, N = 339,224) to investigate BMI-mediated genetic causal pathways to diabetes. We first estimated the causal effect of BMI on diabetes by using four Mendelian randomization methods, where a total of 76 independent BMI-associated SNPs (R^2^ ≤ 0.001, P < 5 × 10^−8^) were used as instrumental variables. It was consistently shown that higher level of BMI (kg/m^2^) led to increased risk of diabetes. We then applied two Bayesian colocalization methods and identified shared causal SNPs of BMI and diabetes in genes *TFAP2B*, *TCF7L2*, *FTO* and *ZC3H4*. This study utilized integrative analysis of Mendelian randomization and colocalization to uncover causal relationships between genetic variants, BMI and diabetes. It highlighted putative causal pathways to diabetes mediated by BMI for four genes.

## Introduction

Diabetes is a long term health condition that affects approximately 1 in 11 adults with rapid increase in prevalence worldwide^1^. Elevated BMI in both children and adults has been consistently found causally associated with the risk of diabetes^2–9^. Genome-wide association studies (GWASs) have identified hundreds of genetic variants, in particular, single nucleotide polymorphisms (SNPs) that are associated with both BMI and diabetes^10–14^, which have induced investigations on the role of BMI-associated SNPs in the development of diabetes^2,15^. However, there was limited data on the pathogenesis of diabetes where BMI was a mediator of the genetic causal effects on this disease.

Publicly accessible large-scale GWAS summary results provide great resources of integrative analyses of disease pathogeneses^16–19^, e.g., Mendelian randomization (MR)^20–22^ and colocalization^9,23–26^. MR is designed for estimating causal effect of an exposure on a disease, where exposure associated SNPs are selected as instruments. These instruments are not necessarily causal SNPs due to linkage disequilibrium (LD). Colocalization explores shared causal SNPs of a pair of traits, whether they are exposures, diseases, or exposure and disease. It was not developed for identifying causal relationship between the traits. Thus, the causal questions addressed by the two approaches are different^27^. Each of the approaches alone is insufficient to investigate exposure-mediated genetic causal pathways to a disease. Very recently, frameworks of integrative analysis by combining MR with colocalization have been developed to identify biological mediators in the causal pathways to various clinical outcomes^28–30^.

Of our particular interest is the underlying causal mechanisms of diabetes. In this study, we aim to (i) couple MR with Bayesian colocalization to explore whether BMI is a mediator in the genetic causal pathways to diabetes; (ii) investigate the performance of two Bayesian colocalization methods, COLOC and eCAVIAR^23,25^. COLOC estimates how likely there is a shared causal SNP in a genetic test region for a pair of traits, by assuming there exists at most one causal SNP in the region for either trait, while eCAVIAR allows for multiple causal SNPs.

**Figure 1:**
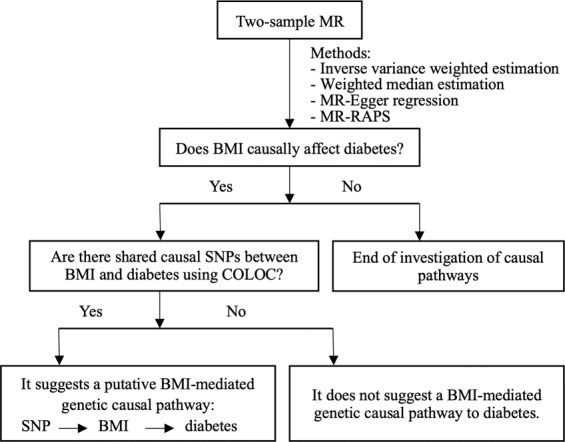
Workflow of our study design. It provides an overview of our investigation of putative BMI-mediated causal pathways to diabetes.

In particular, we exploit the summary results of two independent large-scale GWASs: BMI from the Genetic Investigation of ANthropometric Traits (GIANT) consortium^10^ and diabetes from the UK Biobank^31^ (round 1), by using BMI-associated SNPs as instruments in MR analysis to estimate causal effect of BMI on diabetes. There were several types of diabetes measures in the UK Biobank project. This work focused specifically on “diabetes diagnosed by doctor” with data collected from a touchscreen question “Has a doctor ever told you that you have diabetes?” (https://biobank.ctsu.ox.ac.uk/crystal/field.cgi?id=2443). If there is evidence for a statistically significant causal effect, we then further investigate whether there are shared causal SNPs between BMI and diabetes using COLOC, and therefore gain insights into the underlying mechanisms of diabetes (Fig. 1).

## Results

### Higher BMI causes increased risk of diabetes

We used summary statistics of 76 independent BMI associated SNP instruments (R^2^ ≤ 0.001, P < 5 × 10^−8^) and applied four existing methods (inverse variance weighted estimation, weighted median estimation, MR-Egger regression and MR-RAPS) in our MR analysis. All the MR results consistently showed evidence for a positive causal effect of BMI on diabetes (Table 1), which is agreement with existing literature^2–6^. Table 1 consists of estimated odds ratios (ORs), confidence intervals (CIs) and corresponding *p*-values. The estimated ORs are in the range of (1.038, 1.058) and none of the 95% CIs include value 1. The estimated ORs are fairly precise, with the widest 95% CI (1.015, 1.102) from MR-Egger. Our MR results are strongly suggestive of a causal relationship between BMI and risk of diabetes. For example, in the MR-RAPS method (estimated OR:1.048, 95% CI: (1.042, 1.054)), the odds of an individual being diagnosed with diabetes will increase by 4.8% per 1-SD (or 4.5 kg/m^2^) increase in BMI. The estimated intercept from MR-Egger (estimate: −0.001, 95% CI: (−0.002, 0.001)), not significantly different from zero, suggests that the null hypothesis of zero average horizontal pleiotropic effect is not rejected. The left panel of Figure S1 is scatter plot of the estimated coefficients in the regression analysis of BMI and diabetes on the 76 independent SNPs included as instruments in our MR analysis. The magnitudes of the slopes of the regression lines correspond to the logarithms of the estimated ORs from the first three MR methods in Table 1.

**Table 1:** Estimated causal effect of BMI on diabetes from Mendelian randomization.

MR method	Estimate	95% CI	*p*-value
MR-Egger	1.058	1.015 1.102	9.07 × 10^−3^
(Intercept)	−0.001^*^	−0.002 0.001	0.335
Weighted median	1.051	1.043 1.061	8.18 × 10^−30^
Inverse variance weighted	1.038	1.021 1.057	1.76 × 10^−5^
MR-RAPS	1.048	1.042 1.054	8.27 × 10^−51^

“Estimate” represents the estimated odds ratio, i.e., change in odds of diabetes per 1-SD (or 4.5 kg/m^2^) increase in BMI. ^*^Estimate of “Intercept” in MR-Egger represents the estimated coefficient of horizontal pleiotropy. MR: Mendelian randomization, CI: confidence interval.

In our MR analysis, as shown at the bottom of the left panel of Figure S1, we identified an outlier SNP rs7903146 (within gene *TCF7L2*). This SNP was already found to be an outlier and a horizontal pleiotropic instrument in other studies on BMI-diabetes causal relationships^3,9^, which was supported by previous findings that it was associated with both fasting glucose and BMI^10,32^. We then performed a sensitivity analysis by excluding the outlier. The results without this SNP in Table S1 and Figure S1 (right panel) show that rs7903146 has little impact on the estimated causal effect of BMI on diabetes. For example, in MR-RAPS, the estimated OR increased by less than 0.4% (from 1.048 to 1.052) and 95% CI changed from (1.042, 1.054) to (1.046, 1.057). We further carried out leave-one-out MR analysis. Again, the results changed little in all the MR methods, which suggests none of the SNP instruments influence the MR estimate disproportionately.

### Shared causal SNPs of BMI and diabetes are highlighted for four genes

**Figure 2:**
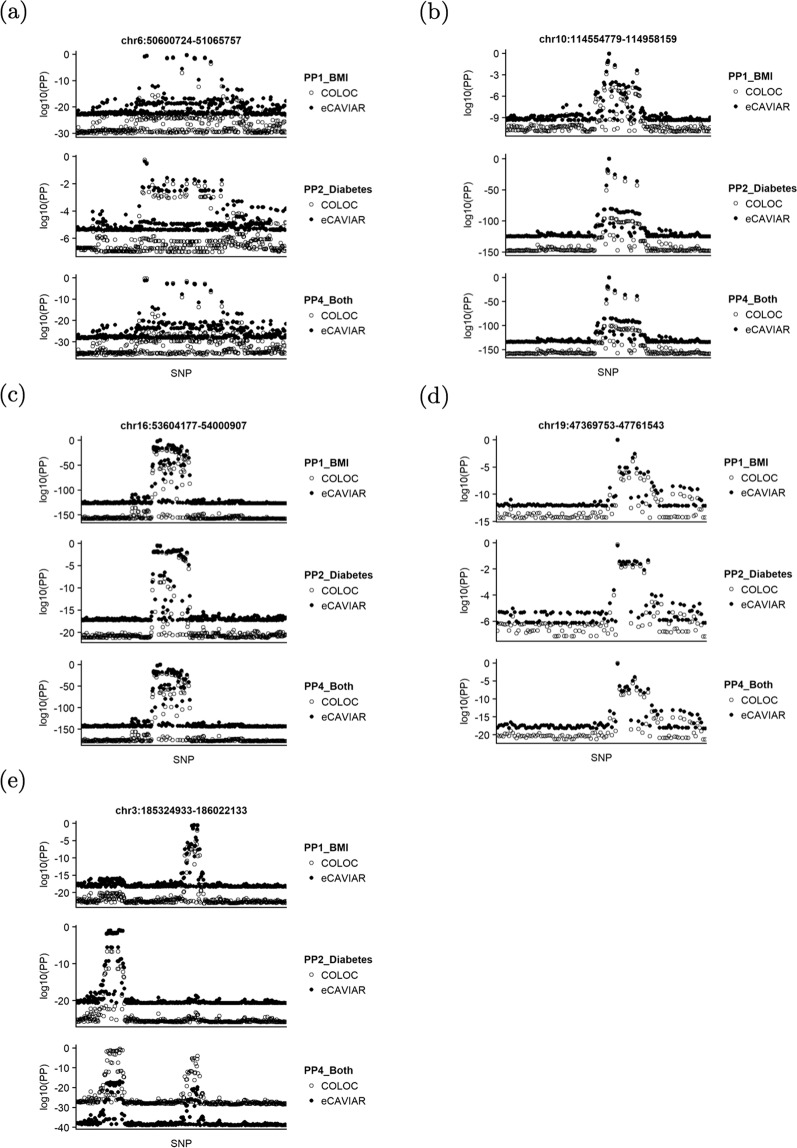
Posterior probability (log10 scale) plot of each of the SNPs causal to BMI only (PP1_BMI, top), to diabetes only (PP2_Diabetes, middle) and to both BMI and diabetes (PP4_Both, bottom), from COLOC (circles) and eCAVIAR (dots) analyses in the five regions showing evidence for one shared or two distinct causal SNP(s). We assume that there exists at most one SNP causal to BMI or to diabetes in each region.

Although we have replicated evidence for a positive causal effect of BMI on diabetes, whether BMI is a mediator on the genetic causal pathways to diabetes is unknown. For such purpose, we used COLOC to test shared causal SNPs between BMI and diabetes. We first included 128 independent SNPs that were associated with either BMI or diabetes (P < 5 × 10^−8^). Each of the SNPs and their neighbours (distance within 200 kb) were then utilized to define a test region. After merging overlapping regions, we tested for colocalization in 118 unique regions using R package coloc, http://cran.r-project.org/web/packages/coloc. Of these unique test regions, four regions in chromosomes 6, 10, 16 and 19 suggest a single causal SNP common to both BMI and diabetes (posterior probability of colocalization PP_4_ > 0.9; Table 2); one region in chromosome 3 suggests two distinct causal SNPs, one for BMI only and the other for diabetes only (posterior probability of distinct causal SNPs PP_3_ = 1; Table 2). In each of the five regions, we further calculated the posterior probability of each SNP being causal to both of the traits (PP4_Both, Fig. 2). Each region/panel comprises three sets of the posterior probabilities on log10 scale at the SNP level: causal to BMI only (PP1_BMI, top), causal to diabetes only (PP2_Diabetes, middle) and causal to both BMI and diabetes (PP4_Both, bottom). For the four regions showing evidence for colocalization (Panels (a)-(d)), SNPs with the maximum PP4_Both are most likely to be causal to both BMI and diabetes, and are thus selected as the candidate causal SNPs. In the region chr3:185324933-186022133 with evidence for distinct causal SNPs (PP_3_ = 1, Panel (e)), two independent SNPs are likely to be candidate causal SNPs.

**Table 2:** Evidence for a shared causal SNP or two distinct causal SNPs between BMI and diabetes shown in five regions.

Region	N	PP_3_	PP_4_	Candidate causal SNP	Chr: position	Gene	Alleles	EAF	B__BMI_	P__BMI_	B__diabetes_	P__diabetes_
chr6: 50600724-51065757	472	0.049	**0.923**	**rs987237**	6: 50803050	***TFAP2B***	G/A	0.09	0.044	1.07 × 10^−30^	0.004	5.67 × 10^−8^
chr10: 114554779-114958159	447	0.001	**0.999**	**rs7903146**	10: 114758349	***TCF7L2***	T/C	0.25	−0.024	1.10 × 10^−12^	0.015	3.72 × 10^−150^
chr16: 53604177-54000907	247	0.026	**0.974**	**rs1558902**	16: 53803574	***FTO***	A/T	0.45	0.084	1.13 × 10^−156^	0.005	1.67 × 10^−22^
chr19: 47369753-47761543	386	0.004	**0.993**	**rs3810291**	19: 47569003	***ZC3H4***	A/G	0.63	0.029	6.35 × 10^−16^	0.003	1.24 × 10^−8^
chr3: 185324933-186022133	112	**1**	0.000	rs9816226^*^	3:185834499	*ETV5*	A/T	0.15	−0.040	6.03 × 10^−24^	0.002	2.88 × 10^−3^
				rs1470580	3:185529174	*IGF2BP2*	A/T	0.29	−0.014	1.03 × 10^−4^	0.006	7.88 × 10^−27^

PP_4_ is the posterior probability of a single causal SNP common to BMI and diabetes in the test region. PP_3_ denotes the posterior probability of two distinct causal SNPs, one for BMI only and the other for diabetes only. N is the number of SNPs included in the test region. Alleles (effect/reference), effect allele frequency (EAF), estimated coefficient (B) and *p*-value (P) are summary results from two independent GWASs: BMI from the GIANT study http://portals.broadinstitute.org/collaboration/giant/index.php/GIANT_consortium_data_files#GWAS_Anthropometric_2015_BMI, diabetes from the UK Biobank study http://www.nealelab.is/blog/2017/7/19/rapid-gwas-of-thousands-of-phenotypes-for-337000-samples-in-the-uk-biobank. * The SNP rs9816226 does not lie within a gene. *ETV5* is its nearest gene.

The results from another Bayesian colocalization method eCAVIAR for the above five regions are also presented in Fig. 2. In Panels (a–d), the results of COLOC (circles) and eCAVIAR (dots) are almost identical in identifying a shared causal SNP between BMI and diabetes. It is clear that if a SNP is most likely to be causal to BMI (highest PP1_BMI), it is also most likely to be causal to diabetes (highest PP2_Diabetes), and consequently most likely to be a shared causal SNP (highest PP4_Both). In panel (e) we observe two distinct causal SNPs, one to BMI and the other to diabetes from both COLOC and eCAVIAR. However, evidence for the two distinct SNPs being most likely to be causal to BMI and diabetes (PP4_Both) from eCAVIAR is much weaker than that from COLOC. Thus, if there exists one shared causal SNP, there is little difference between COLOC and eCAVIAR, at both the region and the SNP levels. In this case, the two approaches can be regarded as alternatives. From Table 2 and panel (e) in Fig. 2, the SNP rs9816226 (nearest gene *ETV5*) is most likely to be causal to BMI only and rs1470580 (within gene *IGF2BP2*) to diabetes only. We have identified the same SNPs that are most likely to be shared causal signals from the two approaches, although at different degrees according to different values of PP4_Both.

Both of the approaches (MR + COLOC and MR + eCAVIAR) have highlighted shared causal SNPs of BMI and diabetes for four genes (*TFAP2B*, *TCF7L2*, *FTO* and *ZC3H4*). In combination with the MR results showing evidence for a causal effect of BMI on diabetes and no horizontal pleiotropy on average, the observed causal effects of these genes on the risk of diabetes might be mediated through changes in BMI.

## Discussion

Interestingly, estimated causal effect of BMI on the risk of diabetes in our MR analysis were smaller but more precise than those reported in the literature. This could be partly explained by (i) the number of instruments. For example, we included 76 instruments in our analysis. However, Fall *et al*. used a single instrument in their study which could not separate mediation from pleiotropy^4^; (ii) sample size. We used GWAS summary data for diabetes from the UK Biobank on the basis of ~ 330,000 individuals which was almost five times the sample size in Corbin’s study^3^; (iii) genotyping platforms. The summary results in this study were from recent GWASs which used more advanced genotyping technology which provides more accurate genotype measures.

MR, COLOC and eCAVIAR use summary statistics from large-scale GWASs. However, as they were designed for different purposes, each of which alone is insufficient to investigate mediators on the genetic causal pathways. We have applied an integrative approach by combining MR with COLOC to investigate BMI-mediated genetic causal pathways to diabetes. The five regions highlighted in COLOC have also been analyzed using eCAVIAR. Our results have consistently shown that if one shared causal SNP is present, then MR + COLOC and MR + eCAVIAR could serve as alternatives. However, where there is evidence for two distinct causal SNPs in a region, eCAVIAR has shown much weaker evidence for each of the distinct SNPs being causal to both BMI and diabetes. This might be because when multiple causal SNPs exist, assuming a single causal SNP results in an underestimation of the posterior probability of a shared causal SNP in eCAVIAR^25^. Thus, if there exists at most one causal SNP to either trait, one may prefer COLOC as it provides relatively robust results. In addition to providing the posterior probability of colocalization, COLOC also computes the likelihood of distinct causal SNPs in a test region, which is another advantage comparing with eCAVIAR when there are two distinct causal SNPs in a test region.

In our analysis, both MR + COLOC and MR + eCAVIAR have consistently shown evidence for causal effects on diabetes mediated by BMI for four genes (*TFAP2B*, *TCF7L2*, *FTO* and *ZC3H4*). Previous studies have suggested that *TFAP2B* is associated with BMI in both Europeans and African Americans^2,11^. This gene has been found associated with waist circumference which is known to be correlated with BMI and may modify the effect of dietary fat intake on weight loss and waist reduction^12,33^. Evidence for its association with diabetes in the UK population and a candidate contributor of the susceptibility to diabetes has also been reported^13^. Thus, the *TFAP2B* gene may play an important role of the underlying mechanisms of diabetes, where BMI is a mediator.

Rs7903146, located in the intronic region of the *TCF7L2* gene, is associated with BMI and diabetes in the European population^10,14^. It was an outlier instrument in our study and in the literature^3^. However, there was little change in estimated causal effect of BMI on diabetes after removing this SNP in our sensitivity analysis. It was identified as a potential pleiotropic but weak instrument. Thus, the MR analysis performed in this study without this SNP provided higher precision in the estimates^3^.

*FTO* is associated with BMI and diabetes^2,33–37^. In addition, rs9939609 in this gene has been identified to affect diabetes through BMI in the UK population^15^. This SNP is in high linkage disequilibrium with the SNP rs1558902 (R^2^ = 0.918) highlighted in our analysis. However, its association with diabetes is reported to be partly independent of its effect on BMI in the Scandinavian population and in east and south Asians^38–40^. Such discrepancy might come from the heterogeneity across populations^41,42^.

Rs3810291 (in *ZC3H4*) has been associated with BMI in European populations^43^. Recent research has further shown that it is associated with both BMI and diabetes in children^2^. Our analysis indicates that this SNP may causally affect diabetes through BMI in the Europeans.

In region chr3:185324933-186022133, there may exist two independent causal SNPs: rs9816226 (nearest gene: *ETV5*) for BMI and rs1470580 (in *IGF2BP2*) for diabetes. Previous GWASs have shown that genetic variation of *ETV5* is predictive of BMI in multiple populations including the Europeans while *IGF2BP2* predictive of the onset of diabetes in European populations and a Chinese Han population^34,44–46^. Thus, *IGF2BP2* may causally affect diabetes but not through BMI.

The MR + COLOC approach in our study assumes that the two non-overlapping samples of the two GWASs are from the same population. We have used the summary statistics from the UK Biobank (British population) and GIANT studies (individuals of all ancestries). One possible limitation of our study would be that if the individuals of the two studies came from difference populations and/or partly overlap, our results may be biased due to violations of the assumption. The unavailability of the individual level data in this study has hindered us from testing the plausibility of this assumption.

Our analysis using MR + COLOC has highlighted, indirectly, four genes with putative BMI-mediated causal pathways to diabetes. These findings, however, need further validation investigations (e.g. mediation analysis using individual level data).

Another limitation is GWAS results of diabetes we downloaded from the Neale lab. They used linear regressions rather than logistic regressions for binary phenotypes, although bias from such model misspecification is not an important issue in our study thanks to a large number of diabetes cases (~ 17,000) and rare variants excluded as instruments.

## Conclusion

In this paper, we have recommended an analytical approach MR + COLOC to investigate causal pathways to diabetes. The causal effects of four genes on diabetes were found mediated by BMI. Both COLOC and eCAVIAR have highlighted the same SNPs that are most likely to be shared causal signals when there is a single shared causal SNP. For a specific study, if we believe there exists at most one causal SNP for either trait or wish to test whether there exists two distinct causal SNPs in a genetic region, COLOC is recommended. If there are multiple shared causal SNPs, one may however prefer eCAVIAR.

The approach applied in the present study takes forward strengths of both MR and colocalization, which can be used as an indirect approach of investigating genetic causal pathways where a mediator conveys the genetic effects. This approach provides new insights into causal mechanisms of diabetes that could be further validated in other studies and ultimately help in the development of new and effective treatments.

## Methods

### Study design and data

Figure 1 depicts the workflow of our study design to investigate putative BMI-mediated causal pathways to diabetes. We used publicly available GWAS summary data of BMI and diabetes. BMI summary data (including major and minor alleles and allele frequencies on 2,554,637 SNPs from 339,224 individuals of all ancestries, estimated effects of allele dose and their standard errors and corresponding p-values) were downloaded from http://portals.broadinstitute.org/collaboration/giant/index.php/GIANT_consortium_data_files#GWAS_Anthropometric_2015_BMI. The same summary statistics of diabetes on 10,894,596 SNPs from 336,473 unrelated individuals from the UK were downloaded from https://www.dropbox.com/s/41q5uj1sa6v99y0/2443.assoc.tsv.gz?dl=0.

### Investigation of causal relationship between BMI and diabetes using Mendelian randomization

**Figure 3:**
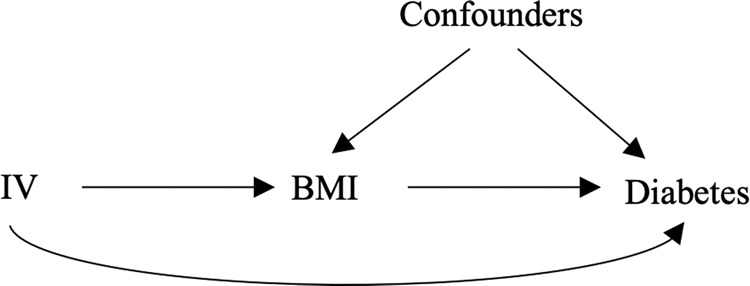
Directed acyclic graph (DAG) of Mendelian randomization analysis using SNPs as instrumental variables (IVs). An IV satisfies three assumptions: (1) it is associated with BMI, i.e., there is an arrow from IV to BMI; (2) it is independent of confounders (both observed and unobserved), i.e., there is no arrow between IV and confounders; (3) it is independent of diabetes conditioning on BMI and confounders (known as no horizontal pleiotropy), i.e., there is no arrow between IV and diabetes. This last assumption, however can be relaxed, for example, in MR-Egger regression. A direct arrow from IV to diabetes is present in this figure because MR-Egger regression was included in our MR analysis.

First, we used MR to test if BMI causally affects diabetes, where BMI associated SNPs were used as the instrumental variables (IVs). The assumptions of MR are represented by a directed acyclic graph in Fig. 3. An IV is: i) associated with the exposure (an arrow pointing from IV to BMI), ii) independent of the confounders (no arrow between IV and confounders) and iii) independent of the outcome, conditioning on the exposure and the confounders (no arrow pointing directly from IV to diabetes). The third condition assumes that there exists no direct effect of the IV on the outcome, i.e., no horizontal pleiotropy^47^, which can be relaxed, for example, in MR-Egger regression. A direct arrow from IV to diabetes is present in Fig. 3 because MR-Egger regression was included in our MR analysis. Of the four MR methods applied in this study, MR robust adjusted profile score (MR-RAPS) was designed to reduce weak instrument bias^29^.

MR requires that SNP instruments are mutually independent. We first filtered the 2,042 BMI associated SNPs (P < 5 × 10^−8^) from the GIANT GWAS results by clumping on the MR-Base platform^48^ to ensure that the instruments in MR analysis were independent of each other. That is, the SNPs in LD (R^2^ ≥ 0.001) were clumped together and only the one with the lowest *p*-value was retained. This led to 76 independent BMI-associated SNPs (Table S2) included as the IVs in MR analysis using four existing MR methods (inverse variance weighted estimation, weighted median estimation, MR-Egger regression and MR-RAPS) in R package TwoSampleMR, https://mrcieu.github.io/TwoSampleMR/. The SNP rs7903146 was detected as an outlier instrument, which was removed in our sensitivity analysis. Leave-one-out analysis was further carried out by leaving one instrument out at a time in MR analysis, to investigate the influence of individual instrumental SNPs on estimated causal effect.

### Identification of shared causal genes between BMI and diabetes using COLOC

Since the IVs are required to be associated with BMI but not necessarily in a causal way, one cannot decide whether such associations are causal. To investigate genetic causal pathways to diabetes, we applied COLOC to detect shared causal SNPs between the two traits: BMI and diabetes. This approach assumes that: (1) in each test region, there exists at most one causal SNP for either trait; (2) the probability that a SNP is causal is independent of the probability that any other SNP in the genome is causal; (3) all causal SNPs are genotyped or imputed and included in analysis. According to these assumptions, there are five mutually exclusive hypotheses for each test region: (1) there is no causal SNP for either trait (H_0_); (2) there is one causal SNP for trait 1 only (H_1_); (3) there is one causal SNP for trait 2 only (H_2_); (4) there are two distinct causal SNPs, one for each trait (H_3_); and (5) there is a causal SNP common to both traits (H_4_). Our primary interest lies in the last hypothesis H_4_ - colocalization. Support for each of the hypotheses is quantified by the posterior probability (PP), denoted by PP_0_, PP_1_, PP_2_, PP_3_, and PP_4_ accordingly. These PPs were calculated from the priors and the approximate Bayes factors. We set the prior probability of each SNP that is causal to either of the traits to 1 × 10^−4^ (i.e. one in 10,000 SNPs in the genome are causal to either trait) and causal to both traits to 1 × 10^−6^ (i.e. one in 100 SNPs in the genome causal to one trait are causal to both traits). We used the GWAS summary statistics of BMI and diabetes to approximate the Bayes factors. COLOC treats trait 1 and trait 2 as two outcomes in no particular order. Thus, it has no capacity for examining relationships between BMI and diabetes.

To define test regions in COLOC, we included the 76 independent BMI-associated SNPs from our MR analysis and 52 independent diabetes-associated SNPs (P < 5 × 10^−8^). Each of these 128 SNPs and their neighbor SNPs (distance within 200 kb on https://genome.ucsc.edu GRCh37/hg19) were used to define a test region. After merging overlapping regions, we tested for colocalization in 118 unique regions that were associated with either BMI or diabetes in COLOC using R package coloc, http://cran.r-project.org/web/packages/coloc.

### Colocalization analysis using eCAVIAR

Next, we applied another Bayesian method eCAVIAR to colocalization analysis. The aim of eCAVIAR is to quantify the likelihood of the number of shared causal SNPs of an exposure and a disease in a test region. This approach assumes that: 1) in each test region, at least one SNP is causal to either the exposure or the disease; 2) the probability that a SNP is causal to the exposure is independent of the probability that it is causal to the disease. It computes the colocalization posterior probability (CLPP) that the same SNP is causal to both the exposure and the disease. This approach allows for multiple shared causal SNPs and is therefore different from COLOC which assumes at most one shared causal SNP in each region. To ensure the results of the two approaches were comparable, we assumed only one SNP is causal to BMI or to diabetes in each of the five regions in eCAVIAR (https://github.com/fhormoz/caviar).

### Ethics statement

Ethical approval was not required for this study that used publicly available GWAS summary statistics.

## Supplementary information

Supplementary information.

## Data Availability

The datasets analysed during the current study are publicly available from http://portals.broadinstitute.org/collaboration/giant/index.php/GIANT_consortium_data_files#GWAS_Anthropometric_2015_BMI by clicking on “Download BMI All Ancestry GZIP” for BMI^12^ and https://www.dropbox.com/s/41q5uj1sa6v99y0/2443.assoc.tsv.gz?dl=0 for diabetes.
