# Supplementary material for: Integrative analysis of Mendelian randomization and Bayesian colocalization highlights four genes with putative BMI-mediated causal pathways to diabetes
Source: Sci Rep. 2020 May 4;10:7476. doi: 10.1038/s41598-020-64493-4 (PMC7198550; doi:10.1038/s41598-020-64493-4)
Supplement: Supplementary file 1 — Supplementary information. [file 41598_2020_64493_MOESM1_ESM.pdf]

# Integrative analysis of Mendelian randomization and Bayesian colocalization highlights four genes with putative BMI-mediated causal pathways to diabetes

Qian Liu,[1,2] Jianxin Pan,[3] Carlo Berzuini,[1] Martin K Rutter,[4,5] and Hui Guo[1,*]

[1] Centre for Biostatistics, School of Health Sciences, The University of Manchester, Manchester, UK

[2] School of Mathematics and Statistics, Xidian University, Xi'an, China

[3] School of Mathematics, Faculty of Engineering and Physical Science, The University of Manchester, Manchester, UK

[4] Division of Endocrinology, Diabetes and Gastroenterology, Faculty of Biology, Medicine and Health, The University of Manchester, Manchester, UK

[5] Manchester Diabetes Centre, Central Manchester University NHS Foundation Trust, Manchester Academic Health Science Centre, Manchester, UK

Corresponding author: Hui Guo

Address: Centre for Biostatistics, School of Health Sciences, The University of Manchester, Oxford Road, Manchester, UK M13 9PL

Tel: +44 161 306 8003 Fax: +44 161 275 5205

E-mail: hui.guo@manchester.ac.uk

**Table S1. Estimated causal effect of BMI on diabetes from four two-sample MR methods when *TCF7L2*(rs7903146) was removed from the variant set**

| MR method | Estimate | 95% CI | | *p*-value |
|---|---|---|---|---|
| MR Egger | 1.056 | 1.037 | 1.075 | $1.86\times10^{-7}$ |
| Weighted median | 1.051 | 1.043 | 1.062 | $3.03\times10^{-28}$ |
| Inverse variance weighted | 1.046 | 1.038 | 1.054 | $5.83\times10^{-30}$ |
| MR-RAPS | 1.052 | 1.046 | 1.057 | $1.12\times10^{-77}$ |

"Estimate" represents the estimated odds ratio, i.e., change in odds of diabetes per 1-SD (or 4.5 kg/$m^2$) increase in BMI. CI: confidence interval.

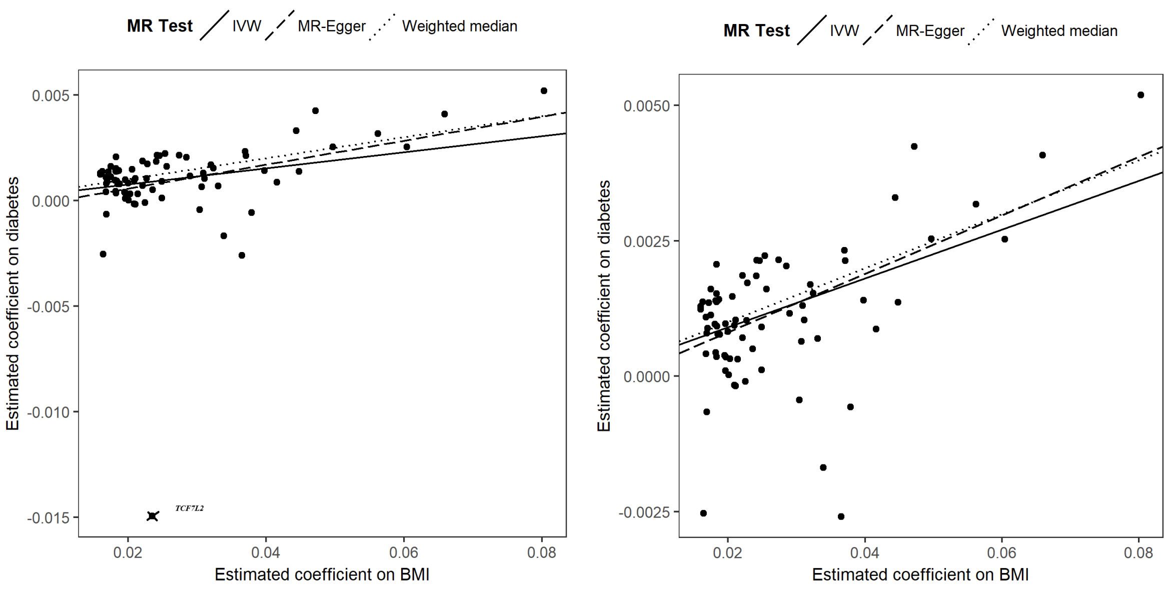


**Figure S1.** Scatter plots of the estimated log odds ratios (slopes of the lines) where the outlier rs7903146 (in gene *TCF7L2*) was included (left panel) and excluded (right panel) from three MR methods: IVW estimation (solid line), MR-Egger regression (long dashed line), and weighted median estimation (dotted line), on the basis of the 76 SNPs selected as instruments in our MR analysis.

**Table S2. Seventy-six independent SNPs used in Mendelian randomization analysis**

| SNP | Chromosome | Position | Effect Allele | Other Allele | Effect Allele Frequency |
|---|---|---|---|---|---|
| rs11165643 | 1 | 96924097 | C | T | 0.425 |
| rs17381664 | 1 | 78048331 | C | T | 0.425 |
| rs2820292 | 1 | 201784287 | A | C | 0.492 |
| rs543874 | 1 | 177889480 | G | A | 0.267 |
| rs657452 | 1 | 49589847 | A | G | 0.417 |
| rs7531118 | 1 | 72837239 | T | C | 0.392 |
| rs7550711 | 1 | 110082886 | T | C | 0.034 |
| rs977747 | 1 | 47684677 | T | G | 0.467 |
| rs1016287 | 2 | 59305625 | T | C | 0.325 |
| rs10182181 | 2 | 25150296 | A | G | 0.500 |
| rs13021737 | 2 | 632348 | A | G | 0.125 |
| rs1460676 | 2 | 164567689 | T | C | 0.783 |
| rs1528435 | 2 | 181550962 | T | C | 0.583 |
| rs17203016 | 2 | 208255518 | G | A | 0.200 |
| rs2121279 | 2 | 143043285 | T | C | 0.117 |
| rs6713510 | 2 | 227034499 | A | G | 0.483 |
| rs7591633 | 2 | 58872058 | G | A | 0.517 |
| rs7599312 | 2 | 213413231 | G | A | 0.708 |
| rs13078960 | 3 | 85807590 | T | G | 0.817 |
| rs1516725 | 3 | 185824004 | T | C | 0.092 |
| rs2365389 | 3 | 61236462 | C | T | 0.658 |
| rs2640017 | 3 | 141335121 | A | G | 0.925 |
| rs6804842 | 3 | 25106437 | A | G | 0.425 |
| rs11727676 | 4 | 145659064 | C | T | 0.075 |
| rs13107325 | 4 | 103188709 | C | T | 0.883 |
| rs13130484 | 4 | 45175691 | C | T | 0.567 |
| rs17001561 | 4 | 77096118 | G | A | 0.833 |
| rs2112347 | 5 | 75015242 | G | T | 0.375 |
| rs7715256 | 5 | 153537893 | G | T | 0.450 |
| rs13191362 | 6 | 163033350 | A | G | 0.800 |
| rs13201877 | 6 | 137675541 | A | G | 0.917 |
| rs2033529 | 6 | 40348653 | G | A | 0.258 |
| rs3800229 | 6 | 108996963 | T | G | 0.692 |
| rs6457796 | 6 | 34828553 | T | C | 0.742 |
| rs9374842 | 6 | 120185665 | T | C | 0.742 |
| rs943005 | 6 | 50865820 | T | C | 0.100 |
| rs1167827 | 7 | 75163169 | A | G | 0.458 |
| rs2060604 | 8 | 76650334 | T | C | 0.558 |
| rs10733682 | 9 | 129460914 | A | G | 0.425 |
| rs1928295 | 9 | 120378483 | C | T | 0.425 |
| rs2183825 | 9 | 28412375 | C | T | 0.292 |

| | | | | | |
|---|---|---|---|---|---|
| rs4740619 | 9 | 15634326 | T | C | 0.533 |
| rs6477694 | 9 | 111932342 | C | T | 0.358 |
| rs17094222 | 10 | 102395440 | C | T | 0.208 |
| rs7899106 | 10 | 87410904 | A | G | 0.950 |
| rs7903146 | 10 | 114758349 | T | C | 0.250 |
| rs10840100 | 11 | 8669437 | G | A | 0.725 |
| rs11030104 | 11 | 27684517 | A | G | 0.800 |
| rs12286929 | 11 | 115022404 | G | A | 0.433 |
| rs2176598 | 11 | 43864278 | T | C | 0.200 |
| rs3817334 | 11 | 47650993 | C | T | 0.550 |
| rs11057405 | 12 | 122781897 | A | G | 0.092 |
| rs7138803 | 12 | 50247468 | G | A | 0.558 |
| rs1006353 | 13 | 28047269 | A | G | 0.283 |
| rs12429545 | 13 | 54102206 | G | A | 0.900 |
| rs1441264 | 13 | 79580919 | A | G | 0.550 |
| rs9540493 | 13 | 66205704 | G | A | 0.550 |
| rs10132280 | 14 | 25928179 | A | C | 0.333 |
| rs7144011 | 14 | 79940383 | T | G | 0.275 |
| rs13329567 | 15 | 68104367 | T | C | 0.217 |
| rs3736485 | 15 | 51748610 | A | G | 0.425 |
| rs12448257 | 16 | 3599655 | G | A | 0.775 |
| rs1421085 | 16 | 53800954 | C | T | 0.450 |
| rs3888190 | 16 | 28889486 | A | C | 0.358 |
| rs4889606 | 16 | 31011183 | G | A | 0.358 |
| rs9926784 | 16 | 19941968 | T | C | 0.792 |
| rs1000940 | 17 | 5283252 | G | A | 0.225 |
| rs12940622 | 17 | 78615571 | A | G | 0.458 |
| rs17066856 | 18 | 58049656 | C | T | 0.133 |
| rs1808579 | 18 | 21104888 | T | C | 0.475 |
| rs6567160 | 18 | 57829135 | C | T | 0.283 |
| rs11672660 | 19 | 46180184 | C | T | 0.825 |
| rs14810 | 19 | 34304903 | C | G | 0.325 |
| rs17724992 | 19 | 18454825 | A | G | 0.692 |
| rs6091540 | 20 | 51087862 | C | T | 0.725 |
| rs2836754 | 21 | 40291740 | C | T | 0.650 |
